# Systematic integration of experimental data and models in systems biology

**DOI:** 10.1186/1471-2105-11-582

**Published:** 2010-11-29

**Authors:** Peter Li, Joseph O Dada, Daniel Jameson, Irena Spasic, Neil Swainston, Kathleen Carroll, Warwick Dunn, Farid Khan, Naglis Malys, Hanan L Messiha, Evangelos Simeonidis, Dieter Weichart, Catherine Winder, Jill Wishart, David S Broomhead, Carole A Goble, Simon J Gaskell, Douglas B Kell, Hans V Westerhoff, Pedro Mendes, Norman W Paton

**Affiliations:** 1 School of Chemistry, The University of Manchester, Manchester M13 9PL, UK; 2 School of Computer Science, The University of Manchester, Manchester M13 9PL, UK; 3 Manchester Centre for Integrative Systems Biology, The University of Manchester, Manchester M1 7DN, UK; 4 School of Computer Science & Informatics, Cardiff University, Cardiff CF24 3AA, UK; 5 Faculty of Life Sciences, The University of Manchester, Manchester M13 9PL, UK; 6 School of Chemical Engineering and Analytical Science, The University of Manchester, Manchester M60 1QD, UK; 7 School of Mathematics, The University of Manchester, Manchester M13 9PL, UK; 8 Department of Molecular Cell Physiology, Vrije Universiteit, de Boelelaan 1085, 1081 HV Amsterdam, The Netherlands; 9 Virginia Bioinformatics Institute, Virginia Tech, Washington Street 0477, Blacksburg, VA 24061, USA

## Abstract

**Background:**

The behaviour of biological systems can be deduced from their mathematical models. However, multiple sources of data in diverse forms are required in the construction of a model in order to define its components and their biochemical reactions, and corresponding parameters. Automating the assembly and use of systems biology models is dependent upon data integration processes involving the interoperation of data and analytical resources.

**Results:**

Taverna workflows have been developed for the automated assembly of quantitative parameterised metabolic networks in the Systems Biology Markup Language (SBML). A SBML model is built in a systematic fashion by the workflows which starts with the construction of a qualitative network using data from a MIRIAM-compliant genome-scale model of yeast metabolism. This is followed by parameterisation of the SBML model with experimental data from two repositories, the SABIO-RK enzyme kinetics database and a database of quantitative experimental results. The models are then calibrated and simulated in workflows that call out to COPASIWS, the web service interface to the COPASI software application for analysing biochemical networks. These systems biology workflows were evaluated for their ability to construct a parameterised model of yeast glycolysis.

**Conclusions:**

Distributed information about metabolic reactions that have been described to MIRIAM standards enables the automated assembly of quantitative systems biology models of metabolic networks based on user-defined criteria. Such data integration processes can be implemented as Taverna workflows to provide a rapid overview of the components and their relationships within a biochemical system.

## Background

Mathematical models are key in systems biology [1] where they typically describe the topology of biological networks, listing biochemical entities and their relationships with one another. The behaviour of a biological system can also be deduced from mathematical models. For example, simulations with a model of a metabolic network can predict how variables in the form of metabolite fluxes and concentrations are influenced by parameters such as an enzyme's maximum catalytic rate. Diverse types of data are required in the construction of mathematical models of biological systems and these are typically held in multiple sources. Information about the metabolites and enzymes involved in a reaction can be found in databases such as KEGG [2] and Reactome [3], as well as in spreadsheet files that have been used to disseminate re-constructed models of metabolism from a number of organisms [4,5]. Curated information on metabolic enzymes and their kinetic properties can be found in various generic and model organism-specific databases including Uniprot [6], SABIO-RK [7] and the Saccharomyces Genome Database (SGD) [8]. Details about metabolites such as their representation in SMILES or InChI formats are available from various databases including ChEBI [9] and PubChem [10].

The assembly of mathematical models of biological systems normally requires a combination of tools [11]. For example, the process may begin by mapping the information for each biochemical reaction and its parameters from its source into a model design tool such as Cell Designer [12]. Network analysis tools such as COPASI [13] can then be used to calibrate parameters by fitting them to a set of experimental observations made from the biological system so that a more accurate response of the model can be attained in simulations [14]. Like other data analysis processes in bioinformatics, the combination of network construction, parameterisation and calibration of systems biology models are *in silico *experiments involving the interoperation of distributed information repositories and computational tools. In systems biology, these *in silico *experiments form an iterative series of model building and hypothesis-driven simulation processes which are employed to understand how biological systems function as a network of biochemical reactions. Such *in silico *experiments can be implemented as workflows consisting of a series of computational tasks that are performed on data from its access, integration and analysis, to the presentation and visualisation of the results. These data processes can be designed and enacted by workflow systems, such as Taverna, which manage the flow of data between computational resources [15,16].

As with other sub-disciplines in the life sciences, a number of data standards in systems biology have been developed for exchanging information within the community. The Systems Biology Markup Language (SBML) is a format which is widely used to represent biochemical reactions in biological models [17]. However, ambiguity in the use of identifiers and names signifying the same entities can impede the exchange and comparison of SBML models. This issue has been addressed by MIRIAM, a project to standardise the Minimal Information Requested In the Annotation of biochemical Models. The exchange of models is facilitated by following the guidelines set out by MIRIAM by annotating components with Uniform Resource Identifiers associated with recognised data types from controlled vocabularies and specific biochemical entities referenced by bioinformatics databases [18]. The popularity of SBML has led to a need to communicate the results of operations performed on models. The Systems Biology Results Markup Language (SBRML) has been proposed as a format that complements SBML by specifying quantitative data in the context of a systems biology model [19]. Several sets of SBRML data can be associated with a model each consisting of a series of values associated with model variables and their corresponding parameter values. SBRML provides a flexible way of indexing simulation results as well as experiment data that come in spreadsheet-like form or multidimensional data cubes to model parameter values according to a reference SBML model.

The adoption and compliance with data standards in systems biology provides an opportunity for mathematical models to be constructed in an automated and systematic fashion. In this paper, we present a workflow strategy for systematically representing and managing the necessary data, and for automating data integration processes in the construction of mathematical models of metabolic networks which adhere to systems biology data standards. Yeast glycolysis is used as an example of a parameterised metabolic network that is constructed by these workflows. These workflows aggregate information from a number of online repositories which are used to disseminate data generated by the Manchester Centre for Integrative Systems Biology (MCISB), and are also available for download for use with other biological systems. Furthermore, the calibration of parameterised models is undertaken by these workflows prior to their simulation using COPASIWS, a web service that provides a programmatic interface to COPASI [13].

## Implementation

**Figure 1:**
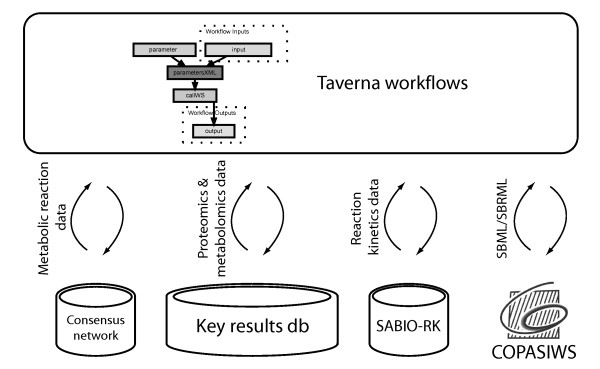
**Schematic diagram showing the generic MCISB informatics infrastructure supporting systems biology studies of metabolic networks**. Metabolite and protein concentrations are stored in a key results database. Enzyme kinetics data are stored in the SABIO-RK database. A web service provides information about the consensus reactions in yeast metabolism. Taverna workflows integrate data from these repositories into mathematical models for analysis by the COPASIWS web service.

**Figure 2:**
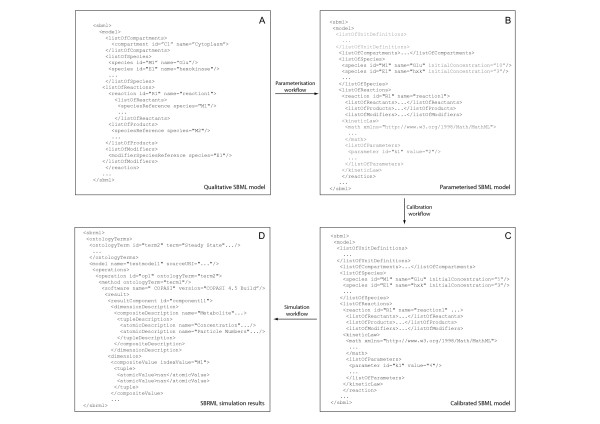
**Schematic view of the data transformations enacted by the systems biology workflows**. (A and B) The modelling workflow generates the qualitative network structure of a metabolic pathway to which parameters in the form of reaction kinetics and starting concentrations (grey) are added by the parameterisation workflow. (C and D) The calibration workflow tunes the parameters in the model based on experimental data; the model is then ready for predictive studies by the simulation workflow, whose results are returned in SBRML format.

**Figure 3:**
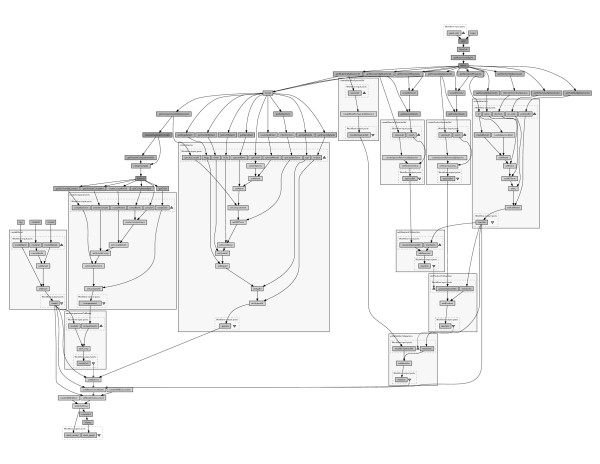
**The workflow used for constructing qualitative models of metabolic pathways in SBML**. Calls to the consensus network web service provide information about the protein, the catalysed reaction and its constituent metabolites for each enzyme from a list of yeast open reading frame numbers. This information is used within nested workflows to iteratively generate components in SBML models using methods from libSBML. An SBML model is produced as the output of the workflow.

**Figure 4:**
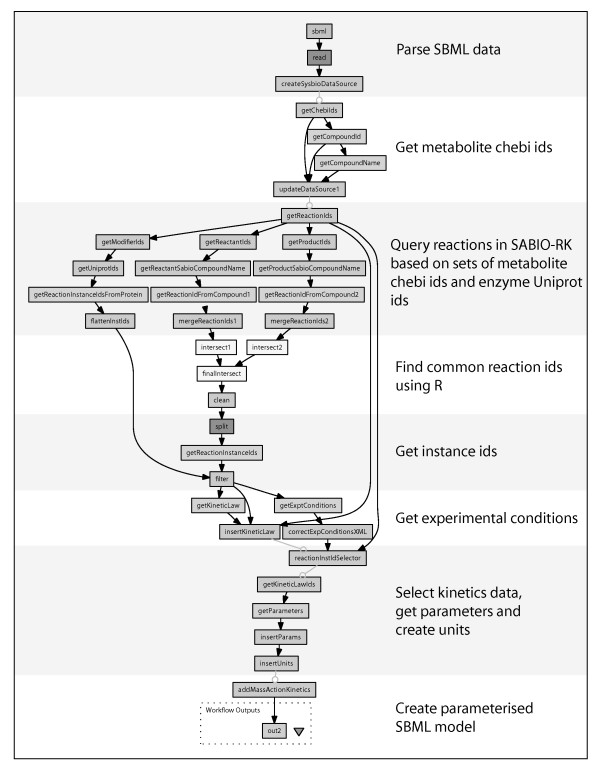
**Model parameterisation workflow integrating experimental data from SABIO-RK and the key results database with a qualitative SBML model**. Quantitative data from SABIO-RK and the key results database were used to parameterise enzymes with their starting concentrations, and reactions with enzyme kinetics.

**Figure 5:**
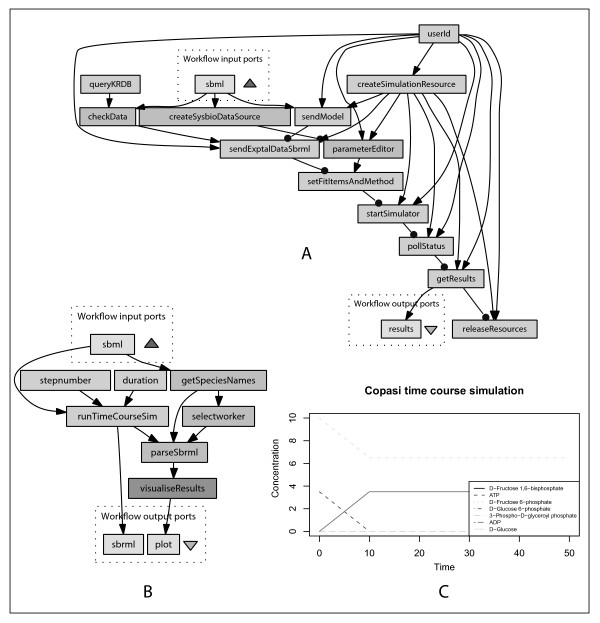
**Two workflows involved in the calibration (A) and simulation (B) of parameterised SBML models using COPASIWS**. (A) The calibration workflow is asynchronous due to the compute-intensive nature of the process. It makes a series of calls to the COPASI web service from the submission and initiation of the calibration task, ending with the retrieval of the results. (B) The simulation workflow is synchronous, making a single call to COPASIWS to parameterise it with input data and waits for the generation of the SBRML file containing the results of the simulation. These results are also plotted as a graph by the workflow (C).

A generic informatics infrastructure for systems biology studies of metabolic networks was developed to support the modelling activities of the MCISB (Figure 1). This infrastructure consists of information repositories to manage data generated in-house including custom databases for storing measurements of the proteome and metabolome from model organisms [20] (Swainston N, Jameson D, Carroll K; A QconCAT informatics pipeline for the analysis, visualisation and sharing of absolute quantitative proteomics data, submitted) (Figure 1). The complex nature of these data, with detailed descriptions of experimental methods and raw measurements, led to a lightweight database being employed for disseminating the key results from proteomics and metabolomics experiments (Figure 1). The key results from such experiments are the quantitative measurements which have direct relevance to biological models and their associated experiment conditions. Kinetic assays measured the parameters and rate at which enzymes catalysed metabolic reactions, and these data were stored in the SABIO-RK reaction kinetics database [7,21]. Information about the reactions catalyzed by these enzymes was curated to MIRIAM standards by a community effort that delivered a consensus genome-scale network of yeast metabolism [22]. These reaction data were stored in an SQLITE database that was deployed as a web service (Figure 1). Computational tools used to process and analyse these data include R and COPASI, a software application for the analysis of biochemical networks which was accessible via its COPASIWS web service [13,23,24]. Workflows supporting the interoperation of the computational databases and tools were designed and enacted using the Taverna workbench (version 2.2) [15]. Four sets of workflows were developed to automate the modelling and simulation of yeast metabolic networks (Additional file 1) (Figure 2, 3, 4 and 5).

### Qualitative modelling of metabolic networks

A mathematical model of a biological system is dependent on information describing its components and their relationships with one another. Workflows were designed which, given a pathway term or a list of yeast enzymes identified by their open reading frame numbers, automatically retrieves information based on these criteria from the yeast consensus network. This retrieved information includes reactant and product metabolites for reactions associated with a pathway term or a metabolic enzyme (Figure 3). The enactment of the workflow integrates this information and produces a qualitative model containing populated lists of compartments, species and reactions in an SBML file (Figure 2A). The procedure of integrating data into an SBML model uses methods from libSBML [25] which have been exposed as workflow components in Taverna using the API consumer application [26] (Figure 3). This workflow also retrieves various annotations for each compartment, species and reaction which are incorporated into the SBML model so that they are semantically-annotated to MIRIAM guidelines [18].

### Parameterisation of qualitative models

A qualitative SBML model has to be parameterised before it can be used in simulating the quantitative systems behaviour of the metabolic network. This requires quantification of the components in the model, as well as their relationships with one another, by parameterising the starting concentrations of metabolite and enzyme species, and their reaction kinetics. Since these data are stored in distributed databases, the process is reliant on integrating the model with quantitative data. To this end, a parameterisation workflow was developed to automate the mapping of proteomics and metabolomics measurements from the key results database onto the starting concentrations of the enzymes and source metabolites (Figure 2 and 4). The reactions catalysed by the enzymes are also parameterised in order to calculate the rate by which metabolite products are converted from reactant metabolites. Reactions are characterised by a kinetic law and associated parameters in SBML, and these are obtained by this workflow from the SABIO-RK database using its web service interface (Figure 2 and 4).

The key to integrating data between model and databases is the MIRIAM-compliant nature of the SBML model that was generated by the qualitative model construction workflow. Metabolite and enzyme species in the SBML model were labelled with identifiers from external databases such as Uniprot or ChEBI. This feature enabled the parameterisation workflow to integrate kinetics from SABIO-RK into the SBML model by querying the database with sets of reactant and product metabolites, and modifier enzymes as described by their database identifiers. In cases where there are multiple reaction instances associated with a given reaction, the parameterisation workflow allows the user to select which particular rate law and kinetics are inserted as part of the workflow. If kinetics could not be found, a mass action rate law is automatically inserted into the reaction in which its rate constants are set to one.

### Model calibration and simulation using COPASIWS

Prior to the use of the parameterised model in predictive studies, the accuracy of its simulations can be improved by calibration with measurements of variables obtained from real biological systems [27]. This process of calibration modifies the parameters of a model until its output matches the given set of real biological measurements. To this end, a workflow was developed to calibrate an SBML model using the parameter estimation feature in COPASI. This feature, along with others in COPASI, has been exposed as web services by COPASIWS [23] (Figure 5A). Calibration of the model with this web service is an interactive process within the workflow, whereby the user defines which parameters in the model and within what range of values they are to be optimized. This was achieved in the workflow through the use of a pop-up window that guides the user through the calibration of the model. The experimental data used to fit the parameters in the SBML model were obtained from the database of key results. In order for parameter estimation to occur, there is a need for the COPASI web service to know how variables in the experimental data map onto entities in the SBML model. This was facilitated by transforming the experimental data into SBRML [19] using a utility web service (Figure 5A).

The resulting calibrated SBML model can be used in simulations for predicting the behaviour of metabolic networks. The COPASIWS provides access to the simulation capabilities of COPASI. This was used in a workflow to derive and solve a series of coupled ordinary differential equations representing the reactions in a SBML model to predict the concentrations of metabolites at various time points (Figure 5B). The results are returned by the COPASIWS in SBRML format and are presented graphically using R as part of the simulation workflow (Figure 2, 5B and 5C).

## Results

The systems biology workflows shown in Figure 3, 4, 5 were evaluated for their ability to generate a quantitative metabolic model of yeast glycolysis. This is a well-understood pathway [28,29] which is being used within the MCISB to assess its different strategies for modelling metabolic systems. Proteomics and metabolomics measurements were made using coupled chromatography and mass spectrometry platforms from samples of *Saccharomyces cerevisiae *grown in continuous culture under turbidostat conditions [30] in a defined minimal medium [31]. The full data set of proteomics and metabolomics measurements was stored in databases implementing PRIDE XML [32] and MeMo [20], respectively. The final concentrations for the metabolites and enzymes in glycolysis were stored in the key results database (Figure 1). Kinetic measurements of two yeast glycolysis enzymes, aldolase (FBA1) and pyruvate decarboxylase (PDC1) were submitted to SABIO-RK for public dissemination.

A qualitative model of glycolysis was generated in MIRIAM-compliant SBML (Additional file 2; sbml_wf1.xml) by the first workflow (Figure 3) which collated data for the glycolytic reactions catalysed by the yeast enzymes shown in Table 1. Parameterisation of the qualitative model (Additional file 2; sbml_wf2.xml) was undertaken by the second workflow (Figure 4) using enzyme kinetics data from the SABIO-RK database. This workflow can insert kinetics for enzymes that were measured by MCISB or use publicly available data where available from SABIO-RK. The starting concentrations of all enzymes were parameterised using data from the key results database, whilst those for metabolites were configured manually. Calibration of the parameterised SBML model (Additional file 2; sbml_wf3.xml) required transforming measurements of metabolite concentrations in the key results database into SBRML format which were then made available, together with the model, to COPASIWS (Figure 5A). Simulations of the calibrated model of glycolysis were then undertaken by the COPASIWS, the results of which were output in SBRML (Additional file 2; sbrml_wf4.xml) and plotted as graphs (Figure 5B and 5C).

**Table 1:** List of enzymes used to generate a model of glycolysis using the qualitative model construction workflow.

Yeast ORF	Function
YAL038W	Pyruvate kinase
YGR254W	Enolase I
YKL060C	Fructose 1,6-bisphosphate aldolase
YKL152C	Tetrameric phosphoglycerate mutase
YFR053C	Hexokinase isoenzyme 1
YLR044C	Major of three pyruvate decarboxylase isozymes
YGR240C	Alpha subunit of phosphofructokinase
YMR205C	Beta subunit of phosphofructokinase
YBR196C	Phosphoglucose isomerase
YCR012W	1 3-phosphoglycerate kinase
YJL052W	Glyceraldehyde-3-phosphate dehydrogenase, isozyme 1
YDR050C	Triose phosphate isomerase

## Discussion

The construction of mathematical models of metabolic networks involving the integration of distributed data can be implemented as Taverna workflows. Automation of these processes provides systematic support for model creation, parameterisation, calibration and simulation, and thus reduces errors or inconsistencies occurring from the manual mapping and tracking of data between information repositories and models. These workflows rely on reaction data which were provided by a community effort to develop a consensus network of metabolism in yeast which met established systems biology standards in the form of SBML and MIRIAM [22].

The construction of models is normally a lengthy and labour-intensive process requiring the manual input of data for each biochemical reaction [33]. This is also true when use is made of applications such as Cell Designer and COPASI which support the modelling of biological systems. Parameterised models can be semi-automatically created using online tools such as SYCAMORE, Systems biology's Computational Analysis and Modeling Research Environment, based on the selection of a set of reactions from SABIO-RK [34], which can then be used in simulations. The way models are constructed in these tools differs from our workflows, which relieve the need for manual entry of data by automatically building an SBML model based on some criteria, such as a list of metabolic enzymes, provided by the user (Figure 3). The resulting SBML model is annotated according to MIRIAM guidelines and this makes it possible for kinetics from SABIO-RK to be systematically integrated into SBML models by the parameterisation workflow (Figure 4). These SBML models provide a starting point for the construction of mathematical models for biological systems, and adherence to standards means that the workflows can consume models developed using other approaches, and that the models produced can be consumed by existing tools.

Previously, the manual assembly of models in systems biology has been preferred due to issues with combining distributed data sources and tools [33]. However, online and downloadable applications can integrate the use of tools and data, for example, the BioModels database [35] can run simulations of the SBML models stored in it via an interface to JWS online [36]. Models constructed using SYCAMORE can also be used in simulations by way of its interoperation with COPASI and ProMOT [34]. A set of Java programs have also been developed by Radrich *et al*., (2010) to integrate data from KEGG and AraCyc to reconstruct qualitative genome-scale models of *Arabidopsis thaliana *[37]. In addition, a Java application called MetaCrop has been developed by Weise et al., (2009) to reconstruct quantitative models of metabolic pathways for plants which can then be simulated using COPASI [38]. Furthermore, a software tool called GRaPe can parameterise the kinetics of reactions and integrate gene expression and protein levels into models for simulation using the SBML ODE Solver in CellDesigner [39]. This current work appears to be a novel application of using computational workflows for the construction, parameterisation, calibration and simulation of metabolic models. The advantage of using workflows is the interoperability of tools and databases by the loose coupling offered through the use of computational resources which have been deployed as web services. Moreover, workflows provide an explicit record of the steps involved in the construction and parameterisation of a model that can be shared for use with the systems biology community.

The enactment of a workflow by Taverna generates provenance to provide a record of the intermediate data that have been integrated into a SBML model which is generally not recorded during the manual construction of models. Using this provenance, we have examined the performance of our workflows. The execution times for both the qualitative modelling and parameterisation workflows were found to increase in a broadly linear fashion with increasing number of reactions (Additional file 3). Using glycolysis as a model test case, the parameterisation workflow took the longest time to execute at 3 min 42 s, followed by the qualitative modelling workflow which took 44.9 s on average. The calibration workflow required approximately 22 seconds to complete, whilst the simulation workflow was the fastest to enact at 6 s. The reason as to why the parameterisation workflow is the bottleneck in these workflows is due to the fact that a large number of queries has to be made to the SABIO-RK database in order to retrieve identifiers to reactions for each metabolite and enzyme for every reaction in the qualitative SBML model. These reaction identifiers are then used to perform a query to identify reaction kinetics stored in SABIO-RK that can be mapped onto reactions in the qualitative SBML model.

Our system for implementing data integration processes as workflows highlighted various data integration issues in systems biology. For example, enzyme kinetics data were not available for every reaction even in a well-studied system such as yeast glycolysis. This required failsafe measures to be undertaken by the parameterisation workflow through the substitution of mass action kinetics in these reactions. Discrepancies were also found between the list of reactants and products in reactions from the consensus model of yeast metabolism compared with those in SABIO-RK. This appears to have arisen from the charge balancing of reactions in the consensus model which caused problems with integrating data from SABIO-RK in our workflows. Inconsistent referencing of metabolites with database identifiers between web services can also hinder the automatic assembly of models. This can lead to anomalous models being built which therefore requires the careful checking of results between each workflow enactment. Future work will enhance the current set of workflows. The criteria against which models can be constructed will be expanded to use, for example, terms from the Gene Ontology [40] so that models for specific biological processes can be generated. A set of workflows will also be developed for the validation of results from systems biology models by their comparison with experimental data.

## Conclusions

Our computational resources and workflows are sufficiently generic that they can be applied to study the metabolic networks of other model organisms. Since there is a dependency of these workflows on reaction information described to MIRIAM standards [41], we have also been instrumental in promoting these efforts through the development of annotation tools [42] and the organisation of community annotation efforts [22]. We are currently participating in ongoing work to deliver a consensus model of human metabolism by consolidation and curation of two existing models [5,43]. It is hoped that the automation provided by these workflows can enable rapid construction and analysis of models in different organisms based on different sets of experimental data, thus enabling more comprehensive experimentation during model development, and more efficient reuse of experimental results.

## Availability and Requirements

All workflows and accompanying documentation are available from http://www.mcisb.org/resources/taverna/sysbio and from ^my^Experiment at http://www.myexperiment.org/packs/107 .The Taverna workbench (version 2.2) can be downloaded from http://www.taverna.org.uk to run workflows which make use of a key results database available from http://beaconw.cs.manchester.ac.uk:8780/mcisbkrdb/and SABIO-RK that is accessible at http://sabio.villa-bosch.de. The COPASI web service is available from http://www.comp-sys-bio.org/CopasiWS/.

## Authors' contributions

PL was responsible for developing the systems biology workflows and writing the manuscript with the help of NWP and DBK. JOD implemented the COPASI web service interface with the aid of PM. DJ designed and created the key quantitative results database with NWP. Proteomics and metabolomics measurements were made by KC, WD and CW, and these data were populated into the key quantitative results database by DJ with the help of NS and IS. NM and JW were responsible for the expression and purification of yeast glycolytic enzymes. Enzyme kinetics were measured by HLM and loaded into the SABIO-RK database by NS. FK, ES, DW, DSB, CAG, SJG, HVW were all involved with the planning of the work along with the other co-authors. All of the authors have read and approved the final manuscript.

## Supplementary Material

Additional file 1 **A compressed zip file containing the systems biology workflows described in this manuscript**. Further information on running these workflows is available at http://www.mcisb.org/resources/taverna/sysbio/index.html. Click here for file

Additional file 2 **A zip file containing SBML and SBRML files that were generated by the systems biology workflows**. Click here for file

Additional file 3 **A MS Word document showing plots of the execution time measurements obtained from the enactment of the qualitative modelling and parameterisation workflows**. Click here for file
