# Supplementary material for: Systematic integration of experimental data and models in systems biology
Source: BMC Bioinformatics. 2010 Nov 29;11:582. doi: 10.1186/1471-2105-11-582 (PMC3008707; doi:10.1186/1471-2105-11-582)
Supplement: Additional file 3 — A MS Word document showing plots of the execution time measurements obtained from the enactment of the qualitative modelling and parameterisation workflows. [file 1471-2105-11-582-S3.DOC]

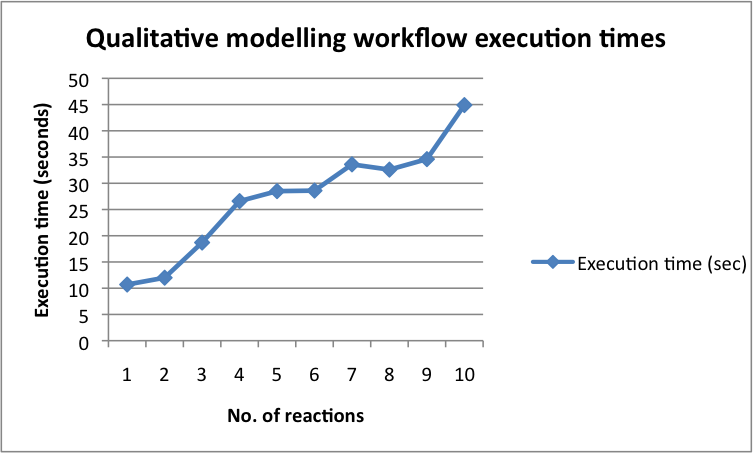


Plot of execution time against the number of reactions to be constructed in a metabolic model for the qualitative modelling workflow.


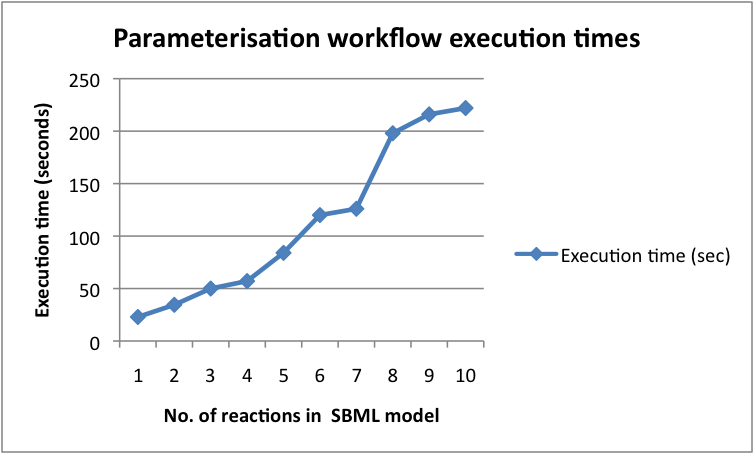


Plot of execution time against the number of reactions to be parameterised for the parameterisation workflow.
